# Revisiting Estrogen for the Treatment of Endocrine-Resistant Breast Cancer: Novel Therapeutic Approaches

**DOI:** 10.3390/cancers15143647

**Published:** 2023-07-17

**Authors:** Nivida Shete, Jordan Calabrese, Debra A. Tonetti

**Affiliations:** Department of Pharmaceutical Sciences, University of Illinois Chicago, Chicago, IL 60612, USA; nshete2@uic.edu (N.S.); jcalab5@uic.edu (J.C.)

**Keywords:** breast cancer, estradiol, estrogen, estrogen receptor, tamoxifen, paradoxical regression

## Abstract

**Simple Summary:**

Estrogen is known to drive the growth of estrogen-receptor-positive breast cancers, the most common breast cancer subtype. Drugs that target the estrogen receptor by blocking estrogen signaling are the mainstay of treatment. These drugs include tamoxifen, aromatase inhibitors and selective estrogen receptor degraders. Paradoxically, prior to the introduction of tamoxifen, estrogen was used to treat patients with breast cancer. This review will highlight insights and the molecular mechanisms of action and provide a vision for the clinical application of this counterintuitive therapeutic approach to improve patient outcomes.

**Abstract:**

Estrogen receptor (ER)-positive breast cancer is the most common subtype, representing 70–75% of all breast cancers. Several ER-targeted drugs commonly used include the selective estrogen receptor modulator (SERM), tamoxifen (TAM), aromatase inhibitors (AIs) and selective estrogen receptor degraders (SERDs). Through different mechanisms of action, all three drug classes reduce estrogen receptor signaling. Inevitably, resistance occurs, resulting in disease progression. The counterintuitive action of estrogen to inhibit ER-positive breast cancer was first observed over 80 years ago. High-dose estrogen and diethylstilbestrol (DES) were used to treat metastatic breast cancer accompanied by harsh side effects until the approval of TAM in the 1970s. After the development of TAM, randomized trials comparing TAM to estrogen found similar or slightly inferior efficacy but much better tolerability. After decades of research, it was learned that estrogen induces tumor regression only after a period of long-term estrogen deprivation, and the mechanisms of tumor regression were described. Despite the long history of breast cancer treatment with estrogen, this therapeutic modality is now revitalized due to the development of novel estrogenic compounds with improved side effect profiles, newly discovered predictive biomarkers, the development of non-estrogen small molecules and new combination therapeutic approaches.

## 1. Introduction

The first report of the counterintuitive benefit of treatment with estrogens in a subset of breast cancer patients was nearly 80 years ago [1]. The efficacy of estrogen treatment was counterbalanced by unfavorable side effects and continued until the introduction of tamoxifen (TAM) into the clinic in the 1970s. Laboratory and clinical trials proceeded to reveal the comparable benefit of estrogens compared with TAM and began to identify patients most likely to benefit from treatment with estrogenic compounds such as 17β-estradiol (E2), diethylstilbestrol (DES), ethinyl estradiol (EE) and fetal estetrol (E4). In the laboratory, long-term estrogen-deprived (LTED) cell lines were established, providing the opportunity to explore the molecular mechanism of the inhibitory effect of estrogen using 2D, 3D and xenograft tumor models. Much has been learned from the establishment of patient-derived xenografts (PDXs) allowing for direct comparison in the laboratory with the patient [2]. However, the inclusion of estrogen into the clinical management of estrogen-receptor-positive (ER-positive) breast cancer has not occurred. The impediment to incorporating estrogens into routine clinical practice is several-fold, including the following: unfavorable side effects, identification of a specific patient population likely to respond and patient and physician acceptability of estrogen use.

In this review, we will discuss the early studies using estrogen treatment prior to the introduction of TAM. This history provides important clues addressing the patient population most likely to benefit from estrogen treatment. The continued dialogue between the laboratory and the clinic is discussed, revealing the importance of estrogen treatment timing following a period of at least 5 years of estrogen deprivation. The clinical trials completed to date and an examination of the side effects associated with these studies provide a perspective on the evolution of estrogenic compounds tested in the clinic. A consideration of potential predictive biomarkers is discussed that can potentially improve the selection of patients most likely to respond to estrogen treatment. The latest advancements in the development of novel estrogenic compounds will be examined in the context of efficacy with improved side effect profiles. New mechanistic approaches are considered, including a non-estrogen small molecule inspired by estrogen’s mechanism of action, an alternating treatment tactic, and a synthetic lethality approach.

## 2. Historical Perspective: Early Use of Estrogen and Estrogen-like Compounds 

Synthetic estrogens for the treatment of advanced breast cancer were first documented by Haddow in a study of 14 pre- and postmenopausal women treated with DES [1]. This seminal study yielded a 36% response rate, paving the way for two significant areas of study: Haddow’s paradox and the rise and subsequent decline of the estrogenic compound DES in breast cancer treatment. Although Haddow was unaware at the time, his paradox refers to the ability of ER-positive breast cancer cells to adapt to a woman’s biological milieu based on the presence, timing, and quantity of circulating estrogen in relation to her menopausal status. In postmenopausal women, those who have undergone oophorectomy, or women with an absence of endogenous estrogen, the presence of estrogen-sensitive cells triggers an inhibitory response upon estrogen exposure. Conversely, in premenopausal women or women with endogenous estrogen, the hormone induces a stimulatory response. This dichotomy elucidates the paradoxical observations in Haddow’s study findings. Another study that same year reported beneficial outcomes when DES was combined with radiotherapy in a small trial of four patients with advanced breast cancer [3]. In the following years, multiple clinicians treated patients with estrogens, androgens and estrogen analogs but could not explain why some patients exhibited dramatic improvement while others showed disease acceleration or no change in disease state [4,5,6,7]. A significantly larger study was published by Kautz involving 944 predominantly postmenopausal women spanning 12 years [8]. Among the 364 patients treated with oral estrogens, mainly in the form of 15 mg daily DES or 3 mg daily ethinyl estradiol (EE), 134 (36.8%) exhibited tumor regression, all of whom were postmenopausal. This study made a crucial observation: an increase in the rate of regression was observed as the time between the initiation of hormonal treatment and menopause lengthened. This observation would later be referred to as the timing hypothesis or gap hypothesis.

### 2.1. The Rise and Fall of DES

DES was first synthesized in 1938 [9] and was initially used to treat advanced breast cancer. By 1946, it was also used to prevent pregnancy complications thought to be caused by insufficient estrogen production [10]. Studies published in 1953 began questioning the therapeutic value of DES in pregnancy and warned of potential dangers [11,12]. Between 1960 and 1970, approximately 25,000 pregnant women per year received DES [13]. By 1971, physicians observed an increase in vaginal adenocarcinoma incidences in young women, which was linked to their mothers’ use of DES during pregnancy [14]. These children were later termed “DES sons” and “DES daughters”. DES sons experienced issues such as epididymal cysts and hypotrophic testes, while DES daughters faced irregular menstrual cycles, infertility, and other reproductive issues. Although DES demonstrated limited effectiveness in preventing pregnancy complications, it maintained its utility in treating breast cancer in postmenopausal women [15]. Estrogen-like compounds served as the primary endocrine therapy for this demographic before the advent of contemporary endocrine treatments, which eventually superseded them. 

### 2.2. Transitioning to Tamoxifen (TAM) and Antiestrogens

Beginning in the 1970s and 1980s, the selective estrogen receptor modulator (SERM) TAM emerged as a preferable alternative due to its reduced side effect profile [16,17,18]. Significant progress was achieved due to a continuous exchange of knowledge between the laboratory and the clinic [19]. The intolerability of the adverse events associated with estrogenic compounds is a significant contributor to treatment failure compared with the better tolerability of TAM [20]. Cumulative side effect data with a prevalence ≥ 5% was extracted and summarized from key studies using EE, TAM, DES, and E2 and presented in Table 1 and Table S1. It is important to note that DES tends to have a more pronounced side effect profile compared to EE and E2, particularly with a higher occurrence of nausea, vomiting, and edema. Whereas TAM showed a similar response rate to DES [21] but had significantly fewer side effects, as shown in Table 1 and Table S1. Side effects were reported in 37% (17 out of 46) of patients using TAM, compared to 54% (36 out of 64) of those using DES. Due to the severity of side effects, 18% (12 patients) discontinued treatment. Common side effects in the DES group included gastrointestinal intolerance, fluid retention, and vaginal bleeding. Subsequent trials, such as the one conducted by Ingle et al. in 1981 [18], yielded similar findings when comparing DES and TAM. A reevaluation of their randomized clinical trial indicated that the 5-year survival rate was significantly better for women in the DES group than those in the TAM group (adjusted *p* = 0.039) [22]. Trials comparing EE to TAM produced results similar to those from the DES and TAM trials. Beex et al. [23] observed that objective remission was achieved in nine out of the 29 patients in the EE group (31%) and 10 out of the 30 patients in the TAM group (33%), demonstrating comparable remission rates between the two treatment groups. In fact, a significant number of studies have indicated that high-dose estrogens (HDEs) show effectiveness equivalent to TAM (reviewed in [24]). 

The development of resistance to TAM, particularly metastatic recurrence following treatment with AIs, has rekindled interest in estrogen-like compounds as potential therapeutic options. Estrogens, such as EE and E2, have comparable response rates for the treatment of advanced breast cancer in postmenopausal women as AIs and TAM [24]. Relevant studies performed in the last 20 years with these compounds as well as novel agents such as E4 and TTC-352, are summarized in Table 2. Among heavily pre-treated patients that received EE and E2, objective response rates (ORR) vary between 0 and 50%, and clinical benefit rates (CBR) vary between 26 and 56%. Fetal E4 has shown promise due to its unique properties because, unlike other estrogens, E4 does not produce active and potentially toxic metabolites. Moreover, it has a long oral elimination half-life, which could enhance its therapeutic effectiveness [25]. Importantly, E4 also presents a more favorable cardiovascular risk profile, making it a potentially safer option for long-term use. Ellis et al. [26] compared a lower dose of E2 (6 mg daily) with a higher dose (30 mg daily) in postmenopausal breast cancer patients with advanced AI-resistant breast cancer. Both doses of E2 resulted in similar CBR (28% and 29%, respectively) and 6 mg E2 dose produced fewer serious adverse events, significantly improving quality of life (QOL) without sacrificing efficacy.

**Table 1:** All-grade adverse events associated with EE, TAM, DES, and E2.

	Ethinylestradiol (EE); *n* = 82	Tamoxifen (TAM); *n* = 184	Diethylstilbestrol (DES) *n* = 117	Estradiol (E2); *n* = 98 * Including E2V
Adverse Events (All Grade)	% of Patients	% of Patients	% of Patients	% of Patients
**Gastrointestinal Issues**				
Gastrointestinal Effects	13%			
Anorexia		6%	10%	1%
Diarrhea		4%	7%	3%
Nausea/Vomiting	34%	17%	56%	20%
**Musculoskeletal Issues**				
Increased Pain/Bone Pain	17%	1%		10%
**Reproductive and Breast Issues**				
Breast Pain				7%
Nipple/Areola Pigmentation	16%			
Vaginal Discharge/Bleeding	17%	1%	9%	13%
Vaginal Spotting	9%	1%		
Withdrawal Bleeding	38%		1%	
**Cardiovascular Issues**				
Congestive Heart Failure			5%	
Fatigue	12%			10%
**Skin, Hair, and Sensory Issues**				
Hot Flashes/Hot Flushes	9%	14%	3%	
**Metabolic and Endocrine Issues**				
Hyponatremia				7%
Edema	16%	5%	38%	
Endometrial Thickening or Uterocervical Enormousness	16%			
**Liver Function Issues**				
Liver Dysfunction/Liver Function Impairment	5%	1%	2%	
**Blood Disorders**				
Leukopenia		5%	1%	

* Table includes values ≥ 5%. Side effects from each estrogenic drug were averaged from similar studies. Please see Table S1 for side effect profile from individual studies [18,21,23,26,27,28,29].

**Table 2:** Use of estrogenic compounds in postmenopausal women (2006–2022).

Treatment (Dose, Number of Subjects, and Duration)	Eligible Patients/Prior Lines of Therapy	Population	Reported Outcomes	References
*n* = 12 Ethinylestradiol: 1 mg daily for 7 to 36 months.	Treated with ≥2 estrogen-depriving therapies and other endocrine therapies.	Aged 49–85 (Mdn 75) ER+ postmenopausal women	ORR = 3/12(25) CBR = 4/12(33)	[30]
*n* = 32 Estradiol: 30 mg daily for evaluation at 24 weeks	Treated with AI and/or one line of chemotherapy.	Aged 39.4–77.7 (Mdn 59.5) ER+ and/or PgR+ clinically postmenopausal women	ORR = 1/32(3) CBR = 9/32(28)	[26] NCT00324259
*n* = 34 Estradiol: 6 mg daily	Treated with AI and/or one line of chemotherapy.	Aged 36.3–83.8 (Mdn 54.7 yrs.) ER+ and/or PgR+ clinically postmenopausal women	ORR = 3/34 (9) CBR = 10/34(29)	[26] NCT00324259
*n* = 18 Ethinylestradiol: 3 mg per day for 3–12 months	Treated with AI and/or endocrine therapies including chemotherapy.	Aged 51–83 (Mdn 63) ER+ clinically postmenopausal women	ORR = 9/18 (50) CBR = 10/18 (56)	[29] UMIN000002831
*n* = 13 Estradiol: 6 mg per day for 3 months.	Treated with ≥1 prior endocrine therapy and progressed thereafter.	Aged 49–85 (Mdn 68) ER+ postmenopausal women	ORR = 3/13(23) CBR = 6/13(46)	[31] NCT01385280
*n* = 19 2 mg estradiol valerate daily for evaluation at 24-weeks.	Treated with a third-generation AI and responded or had stable disease for ≥6 months prior to disease progression.	Aged 49–90(Mdn 67) ER+ clinically postmenopausal women	ORR = 0/19(0) CBR = 5/19(26)	[27] Eudract 2004-000595-14
*n* = 15 TTC-352: 15, 30, 60, 120, 180 mg twice a day for 28-day cycles of treatment until disease progression.	Progressed on two prior lines of hormonal therapy including chemotherapy and mTOR inhibitors. All received a CDK4/6 inhibitor.	Aged 40–62 (Mdn 51) ER+ postmenopausal for ≥1 year or surgically sterile	ORR = 0/12(0) CBR = 2/12(16)	[32] NCT03201913
*n* = 12 Fetal E4; 20 mg, 40 mg or 60 mg per day for 12 weeks.	Treatment failure with tamoxifen and/or AI(s) due to resistance or side effects. Most received prior endocrine, targeted and chemotherapies.	Aged 56–79 (Mdn 70) ER+ postmenopausal women (Natural or surgical) for ≥5 years	ORR = 1/9(11) CBR = 5/9(66)	[25] NCT02718144
*n* = 19 Estradiol 6mg per day for 7–14 days prior to surgery.	Newly diagnosed low-grade, breast cancer without exogenous estrogen exposure	Aged 55.4–75.5(Mdn 67) ER+/HER2- clinically postmenopausal women for ≥ 5 years	ORR = 6/19(32)	[33] NCT02238808
*n* = 19 Alternating estradiol 2 mg orally three times a day for 8 weeks followed by AI for 16 weeks for evaluation at 24-weeks.	Progression on ≥1 antiestrogen or AI-based therapy	Aged 45–80 (Mdn 61) Postmenopausal women with ER+/HER2- breast cancer	ORR = 3/19 (15.8) CBR = 8/19 (42.1)	[34] NCT02188745

Mdn: Median. AI: Aromatase inhibitor. Clinical Benefit Rate (CBR): patients with a CR (Complete response), PR (Partial response), PgR: Progesterone receptor, and SD (Stable disease). Objective Response Rates (ORR): patients with a complete response (CR) plus partial response (PR).

## 3. Optimal Placement of Estrogens in Therapy: Women’s Health Initiative (WHI) and the Million Women Study (MWS)

The WHI and MWS provided significant insight into the role of hormone replacement therapy on the risk of developing breast cancer and the risk of other diseases in women with and without a uterus. Interestingly, women without an intact uterus and who have undergone a significant estrogen gap (>5 years postmenopause) prior to estrogen exposure experienced a lower incidence of breast cancer in the WHI study as well as no increase in incidence in the MWS. These studies were consistent with what was known to date of breast cancer treatment with estrogenic compounds. Further, the molecular mechanisms of estrogen action observed in the laboratory aligned very well with the 5-year gap observed in the WHI and MWS [35]. This dialogue between the laboratory and the clinic has advanced and refined the therapeutic application of estrogen in the breast cancer treatment continuum.

### 3.1. Women’s Health Initiative (WHI) 

A collection of studies published by the WHI in the United States and the MWS in the United Kingdom has yielded significant insights into the stimulatory and chemopreventive properties of estrogens and progesterone on breast cancer in postmenopausal women. The WHI trial was conducted to investigate the uncertain role of estrogen in preventing chronic diseases in postmenopausal hysterectomized women. The aim was to evaluate the effects of common hormone therapy on disease rates, focusing on coronary heart disease and invasive breast cancer incidence. To determine the effects of the two compounds in estrogen-deprived women, the WHI enrolled 10,739 postmenopausal women who had undergone a hysterectomy into a randomized, placebo-controlled trial. Women aged 50–79 received either 0.625 mg of conjugated equine estrogen (CEE) daily or a placebo. The average treatment duration was 5.9 years, but the intervention phase was terminated early due to stroke concerns. Follow-up continued for an average of 6.8 years [36], 10.7 years [37], and 11.8 years [38]. Each follow-up concluded that the use of estrogen was associated with a lower incidence of invasive breast cancer in postmenopausal women who had a hysterectomy. This finding provided strong evidence serendipitously that estrogen causes apoptosis following a period of estrogen deprivation [35].

Conversely, another WHI study was conducted to address the risk–benefit profile of hormone therapy in healthy postmenopausal women with an intact uterus, a question persisting despite years of accrued observational data. Maintaining the same primary focus on coronary heart disease and invasive breast cancer incidence, this study enrolled 16,608 postmenopausal women aged 50–79 with an intact uterus [39]. Participants received either a combination of CEE (0.625 mg daily) and medroxyprogesterone acetate (2.5 mg daily) or a placebo. The intervention phase, with an average treatment duration of 5.2 years for the estrogen plus progestin group, was cut short due to concerns over invasive breast cancer. The hazard ratio (HR) for invasive breast cancer was 1.26, with 166 cases occurring in the estrogen plus progestin group compared to 124 in the placebo group. This WHI study was one of the largest randomized controlled trials to confirm that the combination of estrogen and progestin increases the risk of developing breast cancer. Since halting this study prematurely in 2002, re-evaluation of the data and follow-up studies have concluded that the timing of hormone replacement to within 5 years at the onset of menopause and replacing the progestin component appears to provide symptom relief and reduce breast cancer risk [40]. 

### 3.2. Million Women Study (MWS) 

The Million Women’s Study [41] examined the association between breast cancer risk and the use of the two most common hormonal therapies (estrogen-only and estrogen–progestin formulations), considering factors such as the timing of hormone therapy initiation, duration of use, and the specific agent involved in postmenopausal women. The study recruited 1,084,110 postmenopausal UK women aged 50–64 and asked them to provide prospective information on hormonal therapy use to determine their relative risk (RR) of developing breast cancer. Women were stratified into three groups: current hormonal therapy use, past hormonal therapy use, and never used hormonal therapy. During the studies, cumulative 4.05 million women-years of follow-up [42], 15,759 incidents of breast cancers occurred, 9632 (61%) in women that have ever used hormone therapy, and 7107 (45%) in current users of hormonal therapy. The association between breast cancer risk and the utilization of the two most widespread hormonal treatments, estrogen-only and estrogen–progestin combinations, was assessed by evaluating the initiation timing and duration of hormone therapy usage. The risk of breast cancer was significantly higher for users of both estrogen–progestin (RR = 2.19) and estrogen-only (RR = 1.44) hormonal therapies when used for 5 years or more. Additionally, the risk was significantly greater for current users who began hormonal therapy before or soon after menopause, rather than after a longer gap, for both types of formulations. In estrogen-only users, the risk increased significantly if use started within 5 years after menopause (RR = 1.24), while there was no increase in risk if initiated 5 or more years after menopause (RR = 1.05). For estrogen–progestin users, the risk was significantly higher when therapy began within 5 years after menopause (RR = 2.04) compared to starting 5 or more years later (RR = 1.53). The key takeaway from the MWS with regard to the timing of estrogen exposure is the absence of increased breast cancer risk if administered 5 years after menopause.

## 4. Molecular Mechanisms of Estrogen-Induced Tumor Regression 

Haddow’s paradox describes the seemingly contradictory action of estrogen in ER-positive breast cancer, where estrogen acts as a growth inhibitor instead of a stimulator in response to long-term estrogen deprivation. As previously described, estrogen deprivation can occur if a patient is 5 years postmenopausal or heavily pre-treated with endocrine therapies. Several studies have looked at a variety of preclinical models to determine the molecular mechanism by which estrogen induces apoptosis and tumor regression. These mechanisms include induction of the unfolded protein response (UPR), activation of the extrinsic-death receptor pathway and intrinsic-mitochondrial pathway and down-regulation of pro-survival, cell-growth pathways. Recently, it was reported that estrogen induces DNA damage in long-term estrogen-deprived (LTED) ER+ breast cancer models and offers a potential pathway that can be exploited therapeutically [43]. The establishment of laboratory models of endocrine resistance, including cell lines in 2D and 3D, cell-line-derived xenografts (CDXs) and patient-derived xenografts (PDXs), was fundamental to our understanding of the molecular mechanism of E2-induced inhibition. 

### 4.1. ER+ Breast Cancer Models 

**Figure 1:**
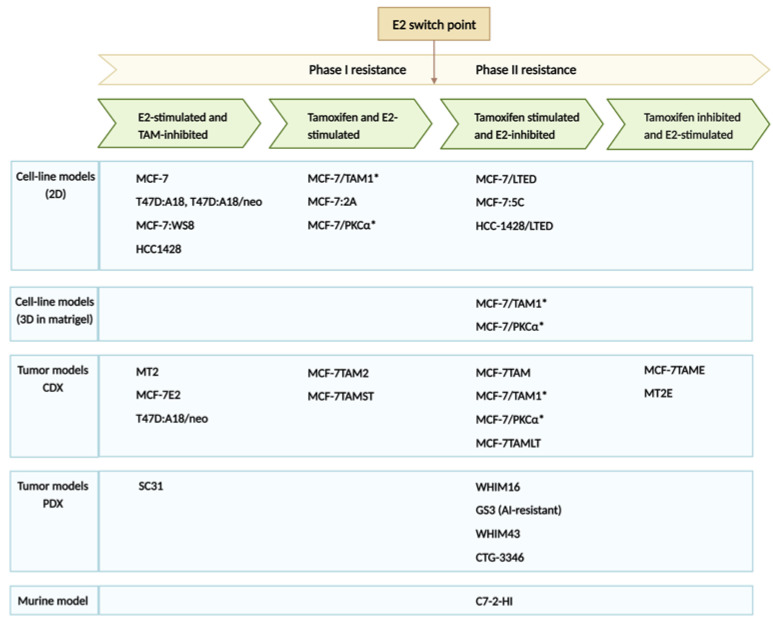
ER positive breast cancer models representing different phases of endocrine therapy resistance [43,44,51,54,56,57,58,59,60,61,62,63,64,66,67,68,69,70,71,72,73,74,75,76,77,78,79,80]. * These cell clones were originally reported to be of T47D origin, through STR testing it was determined they are MCF7. Errata was added to impacted publications. Created with BioRender.com (accessed on 12 July 2023).

The Jordan laboratory has reported comprehensively on the cyclical response of ER-positive breast cancer cell lines and xenograft models, whereby TAM-resistant breast cancer can revert to TAM-sensitivity if exposed to estrogen therapy [44,45,46,47,48,49,50,51]. Numerous biological models representing the cyclical nature of hormone response were invaluable toward elucidating the molecular mechanisms of estrogen-induced tumor regression (Figure 1). Standard endocrine treatment for ER-positive breast cancer includes SERM TAM, AIs (letrozole, anastrozole and exemestane) and SERDs such as fulvestrant, and recently, the FDA approved elacestrant [52,53]. CDX models closely recapitulate the development of TAM resistance as it occurs in patients. Phase I resistance represents the stage of the disease when both TAM and E2 stimulate tumor growth. Between phase I and II resistance, a switch point whereby TAM continues to stimulate tumor growth, whereas now, E2 is inhibitory. After a time, another switch occurs, and TAM now again becomes growth inhibitory, and E2 is once again stimulatory. TAM-resistant breast cancer can be treated with lose-dose E2 for a short duration to reverse resistance, followed by cycling treatment for optimal benefit to patients [44]. The development of breast cancer cell lines and xenograft models has helped to reveal the underlying molecular mechanisms and to create a clinical strategy for the treatment of ER-positive breast cancer. Application of this clinical strategy was recently applied in a small study of 19 postmenopausal patients with advanced breast cancer providing alternating treatment with E2 (2 mg TID) and an AI [34].

LTED cell lines are a surrogate for AI treatment in patients which block estrogen synthesis. Early observations of LTED cell lines showed enhanced expression of ER mRNA and protein levels and transcriptional activation of ER target genes such as c-myc and c-myb [54]. Breast cancer cells adapt to estrogen deprivation by elevating processes such as cell growth/proliferation mediated by the ER, and eventually, this adaption causes hypersensitivity to E2 [54,55]. Osipo et al. described a long-term TAM-stimulated model (MCF-7TAMLT) demonstrating ERα-mediated E2-induced tumor regression [51,56]. Several hormone-independent breast cancer cell lines overexpress PKCα [57]. It was demonstrated that both exogenous and endogenous PKCα overexpression results in hormone-independent growth and TAM resistance [58,59,60]. This breast cancer CDX model generated by implanting PKCα overexpressing MCF-7 cells into athymic mice is growth-inhibited upon E2 treatment requiring ER [58,59,61]. E2 initiates nuclear estrogen-receptor-associated transcriptional activity and alters the levels of many ER-responsive genes [46,62,63]. MCF-7/LTED cells and the WHIM16 PDX model are ESR1 amplified, and these cells express higher levels of ER, ESR1 mRNA, and ER target genes compared with the parental MCF-7 cells [64]. Both the cell line and PDX model are sensitive to the therapeutic effects of E2, which requires high levels of endogenous ER and transcriptional hyperactivation. The cyclical nature of treatment effects is exemplified by the WHIM16 PDX, which ultimately regrows during E2 treatment, indicating the eventual emergence of E2 resistance [64]. The resistance of WHIM16 is associated with ER down-regulation, and the withdrawal of E2 again causes tumor regression [64]. The eventual loss of ER makes the cells surviving E2-treatment stem-like, and later generates E2-resistant ER+ cells [64]. All these cell lines, CDXs and PDX models point toward the cyclical response to E2 and the opportunity to take advantage of the optimal time window for E2 treatment to elicit anti-tumor effects. LTED caused by multiple endocrine therapies in patients may make them susceptible to the therapeutic potential of E2 at early time points. Alternating between estrogen deprivation and E2 treatment at specific “E2 switch points” is a strategy that has emerged from the laboratory [44,45,48,49,50,65,66]. 

### 4.2. Activation of Extrinsic Death-Receptor Pathway and Unfolded Protein Response (UPR)

**Figure 2:**
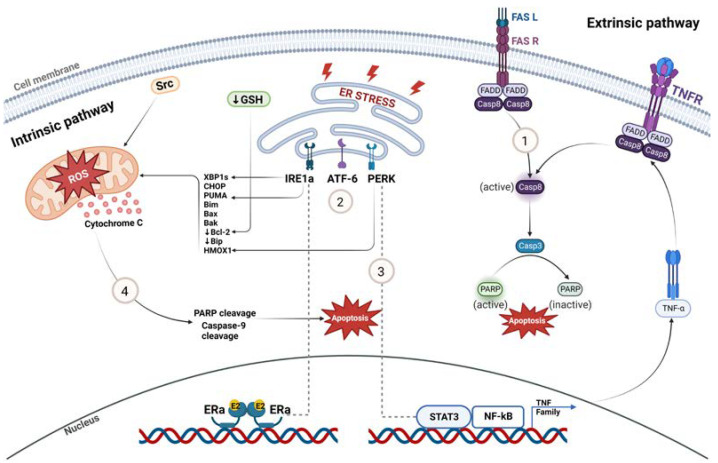
Molecular mechanisms of estrogen-induced tumor regression. (1) Extrinsic death receptor pathway involving FasR-FasL is one of the mechanisms by which estrogen induces apoptosis in LTED breast cancer. (2) E2 rapidly induces endoplasmic reticulum stress and UPR sensors IRE1a, ATF-6 and PERK. (3) UPR-inducer PERK activates NF-κB, which further participates in apoptosis mediated by downstream TNF family member-associated inflammatory proteins in response to E2 treatment in LTED models. (4) Oxidative-stress-induced mitochondrial damage caused by E2 results in apoptosis by increasing the expression of pro-apoptotic proteins and by decreasing the expression of anti-apoptotic proteins. Created with BioRender.com (accessed on 25 May 2023).

Several studies have highlighted the role of the extrinsic death receptor pathway in estrogen-mediated apoptosis involving the Fas receptor (FasR) and Fas ligand (FasL) (Figure 2). Both wildtype and LTED breast cancer cells express FasL, but only LTED cells have elevated levels of FasR and, following E2 treatment, FasL increases, resulting in caspase activation and the induction of apoptosis [67]. In MCF-7TAMLT tumors, ERα appears to mediate E2-induced apoptosis and tumor regression by suppressing the pro-survival/anti-apoptotic factors HER2/neu and NF-κB and inducing the death receptor, FasR [56]. However, anti-Fas antibody does not prevent the apoptosis of MCF-7:5C cells upon E2-treatment, suggesting that this process is not solely Fas-mediated [68,69]. In the CDX model MCF-7/PKCα, E2-induced tumor regression is preceded by the upregulation of Fas/FasL and knockdown of FasR only partially reverses the E2-inhibitory phenotype. Preceding the activation of Fas/FasL, the pro-survival, anti-apoptotic Akt/ Protein Kinase B pathway is repressed, supporting the notion that FasR/FasL action is not solely responsible for E2-induced inhibition [61]. 

Studies analyzing the gene expression profiles of ER-positive tumors after E2 treatment demonstrate the upregulation of multiple pathways related to oxidative and endoplasmic reticulum stress response followed by the activation of the intrinsic mitochondrial pathway and unfolded protein response (UPR), respectively [70]. The WHIM16 PDX and the murine mammary adenocarcinoma C7-2-HI treated with E2 results in increased levels of UPR sensors IRE1α and PERK, and pro-apoptotic proteins CHOP, PUMA and BIM followed by PARP cleavage and apoptosis [63]. Among UPR-regulated proteins, Bip (GRP78) is a protein-folding chaperone that binds and inhibits procaspase-7 cleavage and pro-apoptotic proteins and participates in resistance to antiestrogens [46]. LTED cells have lower basal proteasomal activity and reduced expression of the protein folding chaperone Bip; therefore, it is suggested that upon E2 treatment, low levels of Bip result in IRE1α hyperactivation, leading to apoptosis [63]. 

IRE1α, a UPR sensor regulated by ERα, splices the XBP1 mRNA (an E2-responsive gene) and generates XBP1 transcription factors (Figure 2). XBP1s further control gene expression, fostering protein folding and ER stress. Fan et al. [71] elucidated the role of PERK, a major UPR sensor, in activating NF-κB and NF-κB-dependent genes by increasing its DNA binding capacity after E2 treatment in MCF-7:5C cells. PERK regulates NF-κB and STAT3 DNA binding and increases oxidative stress (HMOX1 levels) [66,71]. There are higher levels of NF-κB in MCF-7:5C than in the parental MCF-7 cells, resulting in the elevated expression of NF-κB-dependent genes, such as TNFα, LTA and LTB [66]. Initially, elevated levels of NF-κB are rapidly suppressed via E2 treatment in MCF-7:5C cells mediated by the complex interaction between C/EBPβ and NF-κB [71]. After 48 h of treatment, PERK plays a role in the late activation of NF-κB and stimulates apoptosis mediated by STAT3 and TNF family-member-associated inflammatory factors (Figure 2) [71].

Other studies demonstrate that the E2 activation of UPR and the consistent, prolonged activation of PERK mediates E2-induced apoptosis by IRE1α inducing endoplasmic reticulum stress-associated degradation (ERAD) of the Akt/mTOR growth-signaling pathway [46,72,81]. Gene expression analysis following E2-treatment of the GS3 PDX model (estrogen suppressed) determined that E2-induced apoptosis is initiated by cell cycle arrest and growth suppression in addition to the upregulation of the tumor suppressor gene, *IL-24* [73]. The downregulation of cell proliferation pathways is confirmed in E2-treated GS3 tumor samples, as evidenced by a decrease in the percentage of cells that progress to the G2/M phase and an increase in the percentage of cells arrested at the G1 phase [73]. Global gene expression profiles of E2-deprived cells (MCF-7:5C and MCF-7:2A) show elevation of JNK (MAPK8) and alterations in many stress-responsive genes involving inflammation, oxidative stress, and endoplasmic reticulum stress [70,74]. LTED cells adapt to estrogen deprivation by elevating activated MAP kinase for DNA synthesis and cell proliferation and are involved in the transactivation of ER [74]. The upregulation of IGF-1R (insulin-like growth factor-1 receptor) and the PI3K/Akt pathway after E2 treatment in MCF-7:5C cells acts as an upstream signal for JNK [66,72]. Interestingly, after 72 h of E2 treatment, the levels of phosphorylated Akt and mTOR (a downstream target of Akt) is reduced in MCF-7:5C cells, suggesting the late disruption of the growth-signaling pathways by E2 [66,72]. As ERAD downregulates Akt, E2 degrades this Akt signaling pathway via the ER stress sensor IRE1α in MCF-7:5C cells [72,81]. This accumulated evidence from multiple laboratories and independent LTED cell lines and tumor models confirm that E2-induced apoptosis is mediated by activation of pro-apoptotic signals (UPR, FasL/FasR) and suppression of cell proliferation pathways, including (Akt/mTOR. MAPK).

### 4.3. Involvement of the Intrinsic-Mitochondrial Pathway in E2-Induced Apoptosis

E2 is reported to induce apoptosis via the intrinsic-mitochondrial pathway with increased expression of pro-apoptotic proteins Bax, Bak, Bim and CHOP, and accompanied by decreased expression of anti-apoptotic proteins Bcl-2 and Bcl-xL (Figure 2) [69]. This pathway results in the release of cytochrome c from the mitochondria to the cytosol, followed by the cleavage of PARP and Caspase-9, initiating apoptosis [68,69]. LTED causes cells to elevate the basal expression of anti-apoptotic Bcl-2 proteins and the suppression of Bcl-2, mediating the pro-apoptotic effect of E2 treatment [50,75]. The elevated expression of tumor-suppressor protein p53 in LTED models in response to E2 is important as it mediates intrinsic apoptosis by transcriptionally turning on pro-apoptotic proteins such as PUMA, NOXA and p21 in LTED cells [63,69]. 

Increased reactive oxygen species (ROS) and HMOX1 mRNA after E2 treatment suggests that E2 has the potential to cause mitochondrial damage by inducing oxidative stress (Figure 2) [66,76,77]. When MCF-7:5C cells were treated with a c-Src inhibitor, E2-induced apoptosis was completely blocked in addition to reducing E-cadherin and increasing N-cadherin expression; characteristic features of epithelial–mesenchymal transition (EMT) [76]. These results indicate that the c-Src tyrosine kinase pathway is required to initiate apoptosis in response to E2 because both the ER and c-Src are upstream regulatory signals of reactive oxygen species (ROS). 

Lewis-Wambi et al. have shown that MCF-7:2C cells are resistant to the apoptotic effects of estrogen, and they have higher levels of glutathione (GSH) and glutathione-related enzymes (GS, glutathione synthetase and GPx2, glutathione peroxidase 2) compared to the parental MCF-7 cells [78]. Anti-apoptotic Bcl-2 protein has an antioxidant property in blocking apoptosis, and its expression levels are coordinated with GSH levels in the cell [82]. The depletion of GSH by L-buthionine sulfoximine (BSO), an inhibitor of the GSH biosynthesis pathway, made resistant MCF-7:2C cells susceptible to E2-induced apoptosis. Apoptosis was correlated with the decreased expression of anti-apoptotic Bcl-2 protein, cytochrome c release, caspase 7 activation, and PARP cleavage (Figure 2) [78]. As suggested by this study, the combination of low-dose estrogen therapy with BSO via the depletion of glutathione levels suggests a potential strategy to treat hormone-resistant ER-positive breast cancer [50,78]. 

### 4.4. Estrogen Induces Receptor-Dependent DNA Damage in ER-Positive Breast Cancer Models

**Figure 3:**
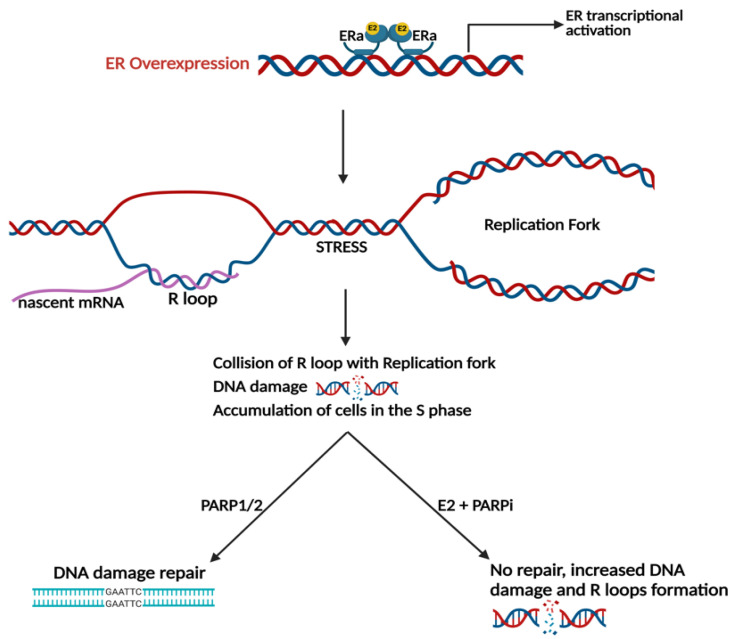
Estrogen-induced DNA damage is ER dependent. E2 treatment induces DNA damage during replication in ER+ breast cancer models via the formation of R-loops can be enhanced via PARP inhibition. Created with BioRender.com (accessed on 25 May 2023).

In MCF-7 cells, E2 induces genomic instability via the formation of ER-dependent R-loops [79]. A recent study conducted genome-wide CRISPR/Cas9 screening and transcriptome profiling of LTED HCC1428 breast cancer cells and determined that E2 induces DNA damage mediated via transcriptional activation of the ER [43]. The molecular mechanism by which E2 induces DNA damage involves the formation of R-loops, which are hybrid DNA-RNA structures formed when nascent mRNA re-anneals to the template DNA strand (Figure 3). R-loops have the potential to cause replicative stress by colliding with the replication fork. As a result, DNA double-strand breaks cause DNA damage, and cells accumulate in the S-phase of the cell cycle. Using both the HCC1438/LTED cell line and WHIM16 PDX tumor models, E2 treatment resulted in the accumulation of cells in the S-phase and an increase in the levels of R-loops confirming DNA damage [43]. The combination of E2 treatment with the PARP inhibitor olaparib enhanced this effect as it prevented PARP1/2 mediated repair of double-stranded DNA breaks [43]. Combination treatment with E2 and a PARP inhibitor to illicit increased DNA damage and tumor regression may prove most effective in the treatment of patients with breast cancer resistant to endocrine therapy.

## 5. Predictive Biomarkers for Response to Estrogens

It is evident that estrogens exert increased effectiveness after a period of estrogen withdrawal, either deriving from menopause, following treatment with AIs or after the emergence of TAM resistance [29,30,83]. While this broad description identifies a patient subpopulation that may gain benefit from treatment with estrogenic compounds, of greater consequence would be a predictive biomarker with better reproducibility. Prospective candidates could be the expression of a single gene or protein (e.g., HER2 or ERα) or a gene signature (e.g., OncotypeDx, Breast Cancer Index, MammoPrint, as reviewed [84]). To date, a few potential single and multigene predictive biomarkers have emerged; while none have been validated, they represent potential clues in identifying a patient subset that may derive benefit from treatment with estrogenic drugs. 

### 5.1. Single Predictive Biomarkers 

*ESR1* amplification is reported to be a predictor of response to estrogen treatment, with evidence from both the laboratory and the clinic [64,85]. The PDX WHIM 16 was engrafted from a patient harboring an *ESR1* amplified tumor, and it was reported that E2 triggered complete tumor regression in both the PDX and in the patient [80]. Another example is a case report of a patient with ER+/PR+/HER2 metastatic breast cancer with *ESR1* amplification derived significant tumor shrinkage in response to E2 [85]. This patient had numerous prior therapies, including several rounds of chemotherapy, endocrine therapy (TAM, fulvestrant, anastrozole), a CDK4/6 inhibitor, everolimus and HER2-directed treatments prior to receiving treatment with 2 mg E2 three times daily. After five months of E2 treatment, the patient experienced a significant decrease in the size of metastatic liver lesions that was sustained for 9 months. *ESR1* amplification is reported to be detected in up to 20–47% of ER+ breast cancer patients [86] and found in patients receiving prior estrogen-reducing treatment from AIs and LHRH agonist therapies [87].

Another potential predictive biomarker may be *ESR1* mutation. It is well-established that 20–40% of metastatic patients treated with AIs develop tumors with *ESR1* mutations [88]. The significance of *ESR1* mutations in determining the efficacy of a variety of breast cancer treatments has been considered [88]. However, the efficacy of estrogenic compounds in this context has not been widely explored. Recently, a study by Schwartz et al. [34] reported the *ESR1* mutational status in 13/19 patients who were treated with alternating E2 and AI. Interestingly, 4/5 patients with *ESR1* mutations experienced tumor regression following E2 treatment, whereas 3/8 patients with wildtype *ESR1* experienced tumor regression. The PDX WHIM 43 was established from a patient with metastatic disease with multiple prior therapies, including AIs. This PDX harbors an *ESR1* D538G mutation and is completely inhibited by E2. [80]. A single patient with multiple prior lines of hormonal and chemotherapy harboring an *ESR1* D538G mutation, when treated with TTC-352, maintained stable disease for over 9 months [32]. Considering that treatment with estrogens is most efficacious following AI treatment, and *ESR1* mutation is prevalent in the metastatic setting, it is reasonable to consider whether patients harboring *ESR1* mutations are responsive to estrogenic compounds. Future clinical trials will be needed to investigate the predictive role of *ESR1* mutations in susceptibility to treatment with estrogenic drugs.

IL-24 was identified as a potential predictive biomarker for E2-induced tumor regression in the GS3 breast cancer PDX model. GS3 was derived from a postmenopausal patient with an AI-resistant brain metastasis [73]. Mice-bearing PDX GS3 tumors treated with E2, revealed via RNA-seq, the upregulation of the tumor-suppressor cytokine, IL-24, a cytokine known to cause cell-cycle arrest and apoptosis. Interestingly, patients harboring tumors with p53 mutations may be resistant to E2 treatment since it was determined that intact p53 function is required for E2-induced regression [63].

Several studies indicate that PKCα is a potential predictive biomarker for the efficacy of E2 in TAM-resistant breast cancer [58,59,61]. PKC is a family of serine/threonine protein kinases involved in several processes, including proliferation, differentiation, apoptosis, and migration [89]. PKCα expression has been connected to more aggressive breast cancers, TAM-resistance and triple-negative breast cancer (TNBC) [90,91,92,93]. The over-expression of PKCα was originally reported as a predictor of TAM resistance and disease aggressiveness [57,90,91] and later determined to be predictive of E2-induced tumor regression in cell-line-derived xenografts (CDXs) and inhibitory in 3D Matrigel and spheroid cultures [58,61]. It was determined that the novel ShERPA, TTC-352, acting as a partial ER agonist, induced regression in PKCα expressing CDXs and the inhibition of cell lines in 3D culture [60]. Preliminary examination of PKCα expression via immunohistochemical (IHC) staining in a Phase I clinical trial with TTC-352 (NCT03201913) found a potential link to response in two patients; however, the very small number of patients and the lack of uniformity in timing and quality of biopsy material diminished the reliance of the findings [32]. The examination of PKCα IHC in a larger patient population in a future Phase 2 trial will be critical to yielding dependable results. 

### 5.2. Multigene Predictive Biomarkers

While the identification of a single predictive biomarker is advantageous, a multigene predictive biomarker panel is preferable. Elevated PKCα expression in both ER+ and TNBC cell lines is reported to initiate a novel EMT pathway, whereby PKCα directly upregulates FOXC2 and acts as a transcriptional repressor of p120-catenin (*CTNND1*), resulting in reduced E-cadherin at the cell membrane [94]. Potentially, PKCα, FOXC2 and *CTNND1* may define a multigene predictive biomarker for E2 response that is superior to PKCα alone. 

A window of opportunity PRe-operative ESTradiOl (PRESTO) trial was conducted, enrolling 19 postmenopausal breast cancer patients with early disease and treated with 6 mg E2 daily for 12 days (Table 1) [33]. Two gene signatures were developed: a pre-treatment or predictive gene signature derived from biopsy cores (PRESTO-30^core^) and a post-treatment signature at the time of surgery (PRESTO-45^surg^). The pre-treatment and post-treatment gene signatures were derived from responders identified as those patients achieving no or low risk of recurrence (ROR) and non-responders from patients with the greatest change in ROR. RORs were derived using Ki67 and BC360 ROR Score (NanoString Technologies). Changes in gene transcription following E2 treatment were used to identify gene expression profiles predictive of response from the PRESTO-30^core^ and differentially altered genes in the PRESTO-45^surg^ in patients that responded. Interestingly, E2 was found to modulate the DREAM complex (dimerization partner, RB-like, E2F and multi-vulval class B), presumably triggering quiescence. These gene expression profiles represent a completely novel mechanism of E2-inhibitory response in a patient population newly diagnosed with early ER+ breast cancer. This trial has identified for the first time a distinct patient population that may derive benefit from E2 treatment.

## 6. Novel Estrogenic Therapeutic Approaches on the Horizon

Over the years, much has been learned about treating specific patient populations with estrogenic compounds, including the determination that lower doses of E2 are as efficacious as higher doses [26]; however, undesirable side effects remain (Table 2). Moreover, the perception of patients and physicians of the counterintuitive use of estrogens to treat ER+ breast cancers greatly diminish the broad acceptability and application of E2 as a useful ER-targeted drug in the therapeutic continuum. A more favorable side-effect profile was achieved with estetrol in the treatment of 12 patients with advanced breast cancer, with five of nine patients showing objective response after completing 12 weeks of treatment [25]. This is an encouraging result, and larger future studies with estetrol are anticipated. 

Another therapeutic approach is based on the activation of UPR, which is identified as a major mechanism triggering estrogen-induced apoptosis. The development of small molecules ErSO and ERSO-DFP to activate a distinct form of UPR called “anticipatory UPR” or a-UPR, were reported to induce ERα-dependent apoptosis in breast cancer cell lines and tumors [95,96]. These molecules cause the shrinking of metastatic ER-positive tumors and it is reportedly tolerated in many species [95]. ERSO-DFP and related derivatives were designed as a modified version of ERSO with enhanced selectivity for ER+ cells [96]. Further characterization of these molecules for drug-like properties and clinical development is expected. 

TTC-352, a selective human estrogen receptor partial agonist (ShERPA) [97], is reported to cause tumor regression via the activation of UPR [62]. TTC-352 has completed a Phase I clinical trial in 15 patients with metastatic breast cancer who were heavily pretreated with numerous prior therapies (nine on average), including cytotoxic chemotherapy. There were no dose-limiting toxicities with a median progression-free survival (PFS) of 58 days, with two best-responders showing PFS of 280 and 309 days. An anticipated Phase 2 clinical trial will determine whether TTC-352 is an effective option earlier in the treatment continuum. 

Recently, a synthetic lethality approach was described, combining E2 with a PARP inhibitor based on the discovery that E2 causes DNA damage (Figure 3) [43]. Because cell cycle progression and DNA replication are necessary for E2 to produce DNA damage, the E2/PARP inhibitor combination is not recommended to be co-administered with CDK4/6 inhibitors [43]. Instead, it is suggested that proper sequencing of treatments can avoid contraindications. The E2/PARP inhibitor combination is effective in cell lines with and without *BRCA1/2* mutations; therefore, the investigators suggest that patients need not harbor *BRCA1/2* mutations to be eligible for this combination treatment. Currently, PARP inhibitors are approved for the treatment of breast cancer patients with BRCA1 or BRCA2 germline mutations [98,99], and perhaps in the future, a new indication will be approved for the E2/PARP inhibitor combination.

## 7. Conclusions

In this review, we have described the history of the counterintuitive application of estrogenic compounds for the treatment of ER-positive breast cancer. Much has been learned since the initial clinical trials performed by Haddow in 1944. The evolution of our understanding of the mechanisms whereby estrogen can cause tumor regression has informed clinical translation. The dialogue between the laboratory and the clinic continued well beyond the time high-dose E2 and DES treatments were abandoned after the introduction of TAM with improved patient tolerability. Research has now shifted to the development of novel estrogenic compounds to reduce harsh side effects, define the therapeutic window when estrogens are inhibitory, and identify specific patient subsets that can derive the greatest therapeutic benefit. More informed decisions about the timing and specific patient response will be available in the future through the validation of predictive biomarkers. Although alternating treatments are currently used to manage metastatic disease, future alternating and combination treatment decisions will be better informed with the completion of ongoing clinical trials. 

## Supplementary Materials

The following supporting information can be downloaded at: https://www.mdpi.com/article/10.3390/cancers15143647/s1, Table S1: Side Effects from Individual Clinical Trials.
